# The CoMPaSS‐NMD Atlas Platform: A Secure Multimodal Infrastructure for Harmonized Data Collection, Exploration, and Future Artificial Intelligence–Driven Stratification in Hereditary Neuromuscular Disorders

**DOI:** 10.1111/ene.70804

**Published:** 2026-11-12

**Authors:** R. Corrias, P. Foszner, A. Nuredini, D. Mendelsohn, A. Töpf, J. Díaz‐Manera, M. Obach, A. Garzo, M. Savarese, J. Verdú‐Díaz, S. Pini, G. La Vella, N. Albano, M. Markiel, L. Piorecki, A. Suwalska, M. Socha, M. Marczyk, S. Bonelli, A. DeAngelis, J. Laporte, G. Caudullo, E. Savino, V. Straub, F. M. Santorelli, J. Polanska, M. P. Scipioni, R. Tupler, B. Schoser

**Affiliations:** ^1^ FINCONS GROUP AG Lugano Switzerland; ^2^ Department of Data Science and Engineering Silesian University of Technology Gliwice Poland; ^3^ Department of Biomedical, Metabolic and Neural Sciences University of Modena and Reggio Emilia Modena Italy; ^4^ Friedrich‐Baur‐Institute at the Department of Neurology LMU University Hospital, LMU Medizin, Ludwig‐Maximilians‐Universität München Munich Germany; ^5^ John Walton Muscular Dystrophy Research Centre, Translational and Clinical Research Institute Newcastle University, and Newcastle Upon Tyne Hospitals NHS Foundation Trust Newcastle upon Tyne UK; ^6^ TECNALIA, Basque Research and Technology Alliance (BRTA) Donostia‐San Sebastián Gipuzkoa Spain; ^7^ Folkhälsan Research Center Helsinki Finland; ^8^ Deep Blue S.r.l. Rome Italy; ^9^ Department of Translational Medicine IGBMC (Institut de Génétique et de Biologie Moléculaire et Cellulaire), Inserm U1258, CNRS UMR7104, University of Strasbourg Illkirch France; ^10^ CeGaT GmbH Tübingen Germany; ^11^ Molecular Medicine and Neurogenetics Unit IRCCS Fondazione Stella Maris Pisa Italy

**Keywords:** artificial intelligence, data management, neuromuscular diseases, precision medicine, rare diseases

## Abstract

**Background:**

Hereditary neuromuscular disorders exhibit marked phenotypic and molecular heterogeneity, which weakens genotype–phenotype correlation and complicates timely diagnosis, prognosis, and patient stratification for clinical trials. The Horizon Europe project CoMPaSS‐NMD aims to develop artificial‐intelligence‐based stratification tools by integrating clinical, genetic, histopathological, and imaging data from clinical reference centers across Europe and the United Kingdom.

**Methods:**

We developed CoMPaSS‐NMD Atlas, a secure, modular web platform for pseudonymized data collection, multimodal data management, and controlled exploration. Clinical, technical, and data‐management experts defined requirements; contributing centers co‐designed electronic clinical and histopathology forms. Clinical phenotypes used the Human Phenotype Ontology (HPO), while imaging, histopathology, and sequencing data followed international standards.

**Results:**

The platform supports role‐ and attribute‐based access, pseudonymized patient registration, metadata management, structured clinical data entry, MRI and histopathological uploads, and a dedicated genetic‐sequencing workflow. Cohort‐level exploration is driven by structured metadata and HPO terms, enabling hierarchical phenotype navigation, ontology‐aware data navigation, and cross‐domain queries spanning clinical, genetic, imaging, and histopathological features. Users can download raw files in their original formats, along with structured metadata reports in CSV or PDF, according to the permissions defined for data protection.

**Conclusions:**

The Atlas provides a foundation for harmonized, standardized prospective data collection and for building large datasets for artificial intelligence‐ and machine learning‐based patient stratification. With secure governance, traceability, and multimodal integration, it is designed to support diagnostic and stratification research in hereditary neuromuscular disorders; clinical validation is ongoing.

## Graphical abstract

Clinical, imaging, histopathological, and genetic data on hereditary neuromuscular disorders remain fragmented across European centers. The CoMPaSS‐NMD Atlas Platform links these data within one pseudonymized record per patient, using HPO‐encoded phenotypes, standard file formats, role‐ and center‐based access control, and Data Access Committee–governed raw‐file access. Deployed in production and technically verified, it enables ontology‐aware cross‐modal cohort queries and provides the foundation for future AI‐based stratification; clinical validation is ongoing.

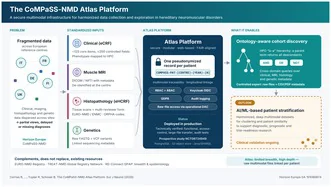

## 1 Introduction

Hereditary neuromuscular diseases (HNMDs) are progressive genetic disorders that cause muscle weakness, atrophy, long‐term disability, impaired participation, and early death [1, 2]. Diagnosis relies on costly investigations and molecular assessments that often remain inconclusive; without a final diagnosis, prognosis is uncertain and patients cannot be enrolled in targeted trials. A central obstacle is data fragmentation: clinical, genetic, histopathological, and imaging data typically remain dispersed across centers and countries, providing only a partial view of disease complexity and contributing to persistently low diagnostic rates.

The Horizon Europe project CoMPaSS‐NMD (Computational Models for New Patient Stratification Strategies of Neuromuscular Disorders; launched in May 2023) develops methods for precision diagnosis and patient stratification in HNMDs [1, 2, 3]. It integrates standardized clinical, genetic, histopathological, and magnetic resonance imaging (MRI) data to build validated multidimensional datasets and artificial‐intelligence (AI) and machine‐learning (ML) tools—including high‐dimensional clustering and federated‐learning‐inspired approaches—for stratification and diagnosis [1, 2]. Prospective data collection is registered with ClinicalTrials.gov (NCT06734949) [4]. Specialized centers in Italy, France, Germany, Finland, and the United Kingdom contribute data through European rare‐disease networks [1].

A central outcome is the CoMPaSS‐NMD Atlas Platform, a FAIR‐aligned digital infrastructure for gathering, organizing, and exploring multimodal HNMD data within a controlled research environment [2]. Designed not as a mere repository but as an operational environment for prospective data entry, file upload, metadata linkage, access control, controlled export, and cohort identification, it aims to support harmonized data collection, more accurate phenotyping, and improved stratification, and to make curated data available to the community. This manuscript describes its design, implementation, and current capabilities.

## 2 Methods

The Atlas was designed through a structured requirements process that involved clinical, technical, and data‐management experts from all centers. For each domain (clinical, imaging, histopathological, genetic), working groups defined minimum and extended variables for patient characterization and downstream analysis, drawing on established standards: the Human Phenotype Ontology (HPO) for phenotype encoding [5], EURO‐NMD neuromuscular pathology guidance for histopathology [6], DICOM for imaging, and FASTQ/VCF for sequencing—chosen to ensure interoperability with external rare‐disease resources and future federated analysis. The electronic clinical (eCRF) and histopathology (eHCRF) case report forms were co‐designed with clinicians, validated against real‐world workflows, and refined before deployment; free‐text input was minimized throughout to maximize analyzability and machine‐readability.

### 2.1 Platform Architecture

The Atlas is a modular, browser‐based platform that requires no local installation and is organized into three interconnected layers spanning the data lifecycle. The user interface layer provides purpose‐built, module‐specific views for each task—patient registration, clinical forms, imaging and histopathology upload, genetic data, record review, and export—so users see only functions relevant to their role. The data storage layer separates structured information (pseudonyms, clinical variables, phenotypic terms, file metadata, permissions), held in a relational database optimized for search and export, from large biomedical files (MRI series, histopathology images, sequencing outputs), held in a dedicated file repository; this separation enables rapid cohort‐wide querying while preserving a traceable link between each file and its pseudonymized record. The role control layer verifies identity, manages sessions, and enforces access rules in concert with the model in Section 2.2. Full technical detail is provided in Box 1. For a user walkthrough, see Video S1.

BOX 1 **Technical focus box.**

**Table:** 

The interface is built with React (styled with Tailwind CSS and the HeroUI library), with each data module implemented as an independent component set for iterative extension. The application server runs on Node.js, exposing a RESTful API over HTTPS with JSON payloads; every request is validated against the authenticated user's token claims, enforcing authorization at the service layer. PostgreSQL is the structured store, holding pseudonyms, CoMPaSS identifiers, clinical variables, HPO mappings, file metadata, user attributes, access‐control rules, and patient–data linkages with referential integrity across modules. Large biomedical files (DICOM, NIfTI, BigTIFF whole‐slide images, FASTQ/VCF) reside in a MinIO‐compatible, S3‐API object store, decoupled from the database so that files from a few megabytes to tens of gigabytes do not affect query performance; presigned‐URL direct upload bypasses the application server for efficiency. Authentication uses Keycloak (OpenID Connect / OAuth 2.0), issuing signed JWT tokens whose role and attribute claims enforce RBAC and ABAC without per‐request database lookups. HPO‐based querying uses a semantic layer built on Apache Jena with an Apache Fuseki SPARQL endpoint: patient phenotypes are modeled as RDF triples over the HPO directed acyclic graph, so a parent‐term search automatically returns all patients annotated with any descendant term, and cross‐domain SPARQL queries combine clinical, imaging, histopathological, and genetic metadata in a single federated query. The platform is deployed on infrastructure at the Silesian University of Technology (Gliwice, Poland), with separate development and production environments, HTTPS enforced throughout, and a modular containerized architecture that supports independent scaling and updating of components.

### 2.2 Access Control and Data Governance

Access combines Role‐Based (RBAC) and Attribute‐Based (ABAC) control. RBAC defines what actions a user may perform; ABAC restricts which data they may act on, the key attribute being clinical‐center affiliation, so users with the same role operate only on their own center's data. Three roles are defined: Clinical Centre Contributor (registration, data entry and editing, upload, and review, limited to the user's own center); Researcher (browse, filter, and access structured metadata to identify cohorts, but no upload and no raw‐file download without a formal Data Access Committee [DAC] request evaluating scientific justification and ethical compliance); and Technical Administrator (unrestricted access for maintenance and deployment, with all actions logged). DAC governance is outlined in Section 3.3.

### 2.3 Pseudonymization and the CoMPaSS Code

Pseudonymization is applied at registration: only the minimum information required to initialize a record is collected, and directly identifying data (name, address, health number) are not entered or stored. Date of birth is recorded as a clinical variable at month‐and‐year resolution only (the day is set to 01), because age at onset, age at each assessment, and disease duration are required for natural‐history analysis and stratification; it is therefore visible in the record and exploration views (Figure 5; Video S1), is handled as pseudonymized personal data under the GDPR, and is subject to the same role‐ and center‐based access rules as all other clinical variables. Two identifiers are generated automatically. The CoMPaSS Code is a structured, human‐readable identifier for every record and upload, formatted COMPASS‐[TYPE]‐[CENTRE]‐[YEAR]‐[COUNT] (TYPE = PAT, CLI, MRI, HIS, or GEN; e.g., COMPASS‐PAT‐000‐2026‐0000), interpretable at a glance yet containing no personal information. The CoMPaSS ID is a long, random, machine‐oriented identifier serving as a unique technical reference. Pseudonymization rather than full anonymization is deliberate: it preserves the capacity to correct errors, update longitudinal records, honor consent withdrawal, and return clinically relevant findings, while the Atlas itself never holds the re‐identification key. The mapping to personal data is stored only at the contributing center, outside the Atlas; for all other users, the identifiers are opaque. All subsequent clinical, MRI, histopathology, and genetic entries link to the pseudonymized record, ensuring full multimodal traceability.

### 2.4 Clinical Data Module

Structured, prospective clinical data are collected through an interface aligned with the project's electronic clinical form, built around neuromuscular center workflows. It comprises approximately 125 core items across domains including demographics, medical and family history, neurological examination, functional parameters, MRC scoring, serum creatine kinase, and disease‐specific features (onset, progression, ambulatory status, cardiomyopathy, respiratory involvement), expanded digitally into over 200 controlled input components; free‐text is minimized to improve reliability, comparability, and statistical evaluation. Relevant parameters are mapped to HPO identifiers, so each phenotype is stored both as a structured variable and as an internationally recognized ontology term. This dual representation preserves clinical granularity within a standardized semantic framework, designed to enable high‐resolution genotype–phenotype and phenotype–phenotype analyses, interoperability with external resources, and cross‐center cohort comparison (Figure 1).

**FIGURE 1:**
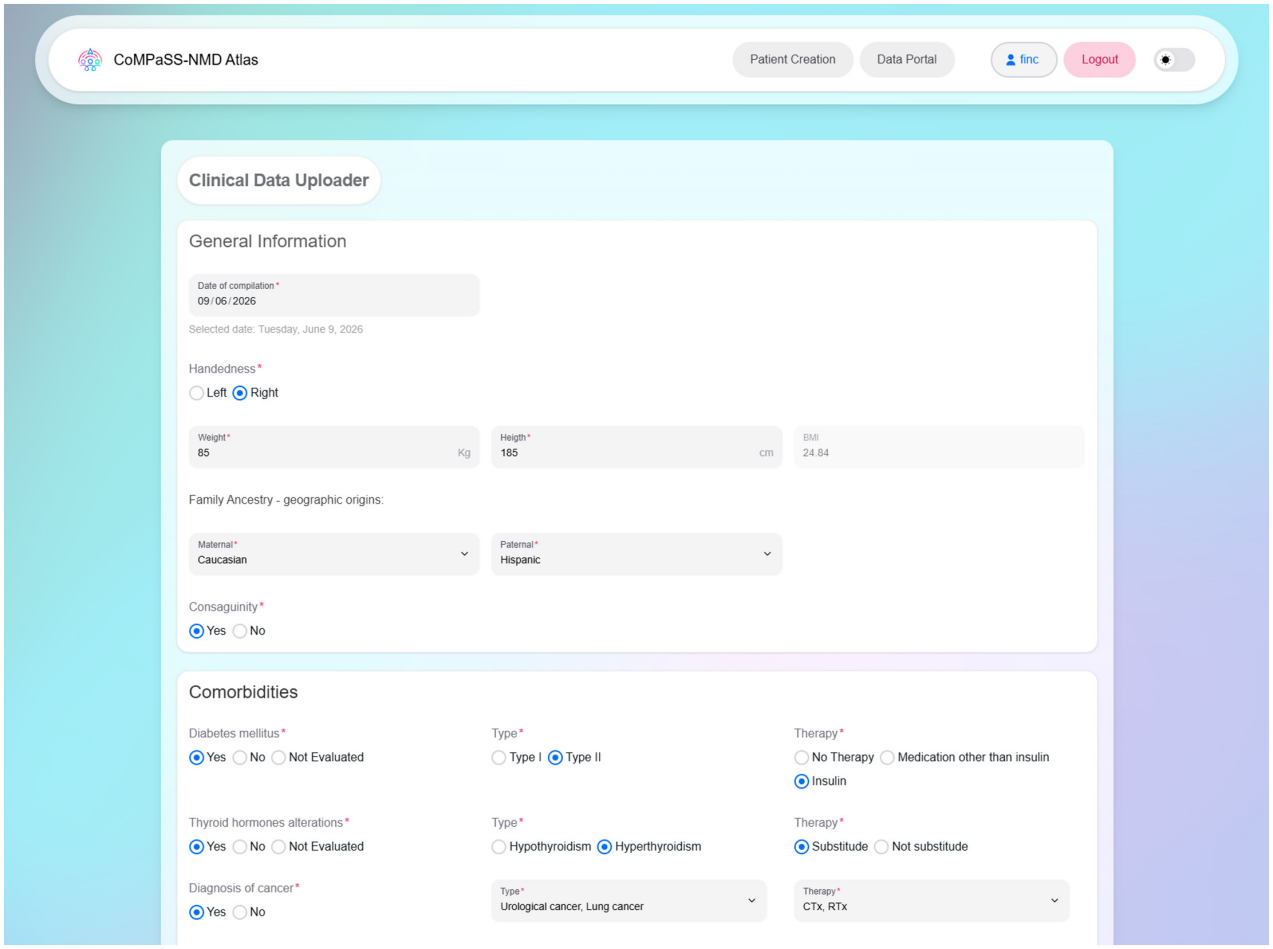
Clinical data module. Screenshot of the structured clinical data‐entry interface (electronic clinical case report form, eCRF).

### 2.5 MRI Data Module

Raw imaging is uploaded in DICOM, NIfTI, or ZIP formats with metadata; files must be anonymized at the center (headers stripped of identifiers) before transfer. Four body regions are supported (lower body, lower leg, whole body, and other muscle groups). Large files transfer directly to the secure repository, with metadata stored in the database and linked to each pseudonymized record. Raw‐file download is governed by the DAC, whereas metadata reports (muscle groups, sequences, acquisition date, center) can be exported as CSV or PDF and remain searchable and filterable (Figure 2).

**FIGURE 2:**
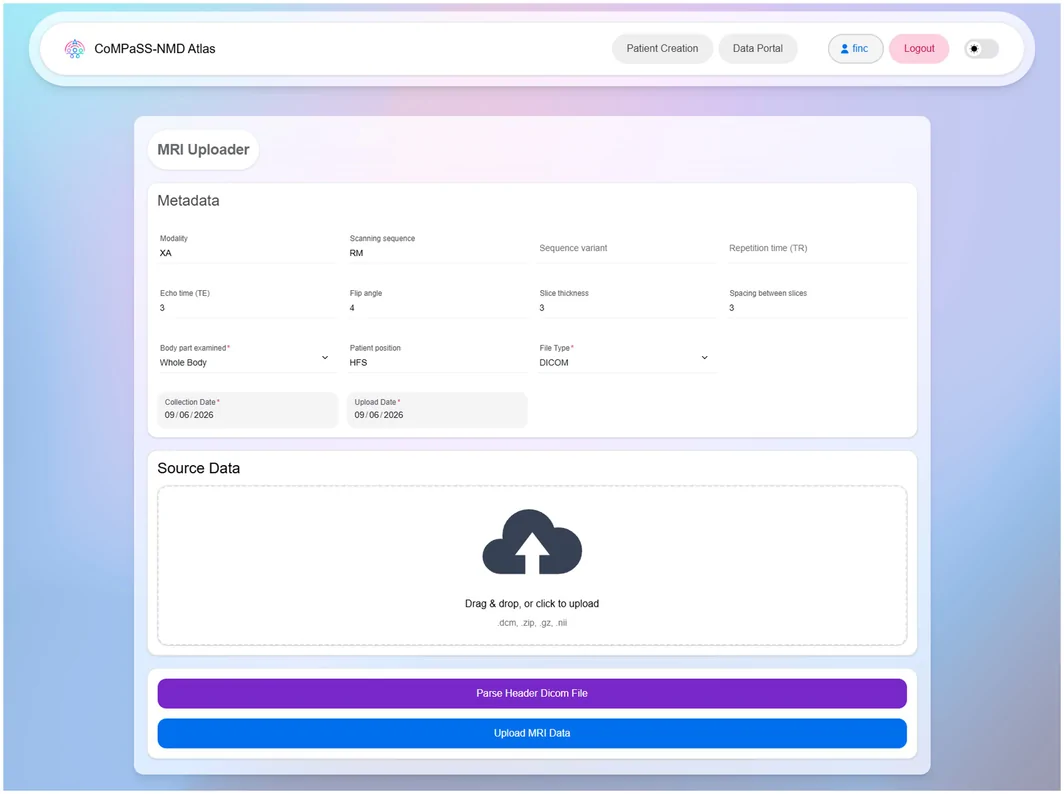
MRI data module. Screenshot of the MRI upload interface for raw imaging data (DICOM, NIfTI, or ZIP archives) and associated metadata.

### 2.6 Histopathological Data Module

Tissue‐scan files, preview images, and metadata are registered through the electronic Histology Case Report Form (eHCRF), a central component of the consortium's eCoreCRF dataset. The eHCRF combines an objective metadata section (sampled muscle, biopsy method, staining, magnification) with a structured assessment capturing pathological features—including central or internal nuclei, nemaline rods, rimmed or round vacuoles, ragged‐red fibers, cores and minicores, moth‐eaten and lobulated fibers, tubular aggregates, inflammatory infiltration, and fiber morphology [6]. It builds on ENMC recommendations, EURO‐NMD pathology guidance, and harmonized Common Data Elements, relies mainly on closed fields, and can link the final diagnosis to ORPHA codes. Each sample may carry one or more independent histological evaluations, allowing interpretive, multi‐reviewer assessment alongside the structured data. Authorized users review previews, edit metadata, and download raw data with CSV or PDF reports. The module thus supports collection, consultation, structured evaluation, and secure export of histopathology data, and lays the groundwork for histopathology‐specific image descriptors (Figure 3).

**FIGURE 3:**
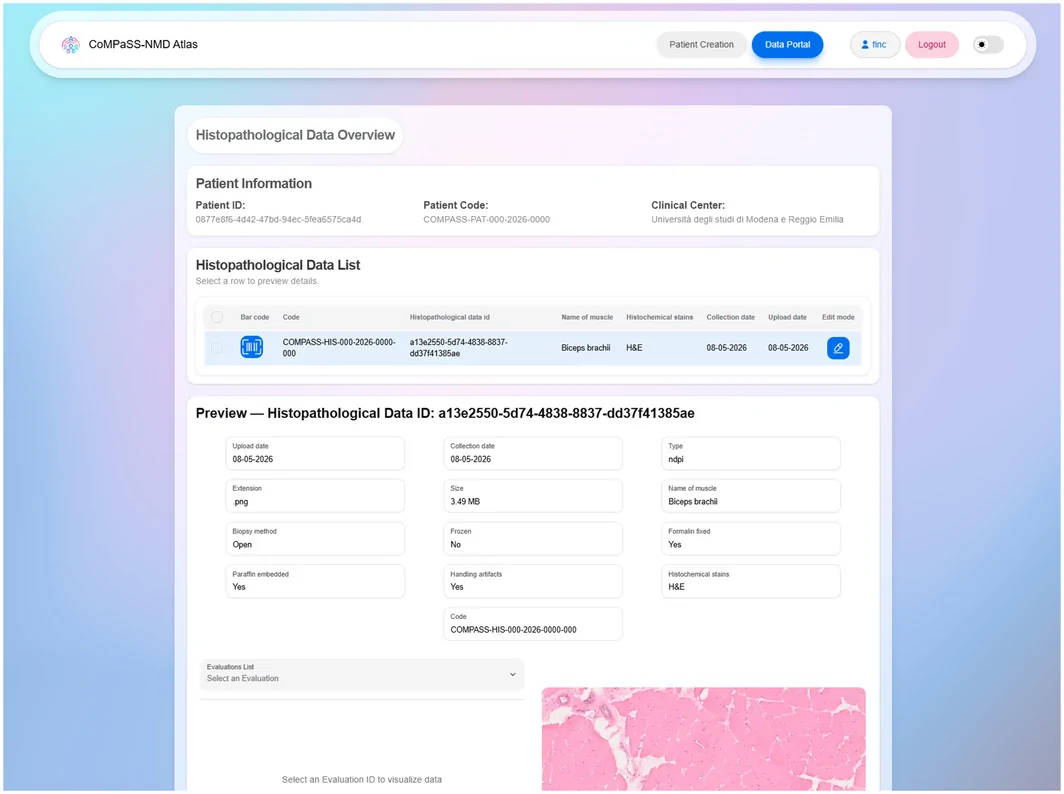
Histopathological data module. Screenshot of the histological data overview, showing uploaded tissue scans, preview images, and structured biopsy metadata.

### 2.7 Genetic Data Module

Whole‐exome or whole‐genome data are integrated by direct upload of raw sequencing outputs (FASTQ) to secure object storage, with processed variants uploadable as VCF. Associated metadata keep sequencing outputs linked to the correct pseudonymized patient and harmonized across modalities. Authorized users can download raw data and variant lists in their original formats together with CSV or PDF metadata reports (Figure 4).

**FIGURE 4:**
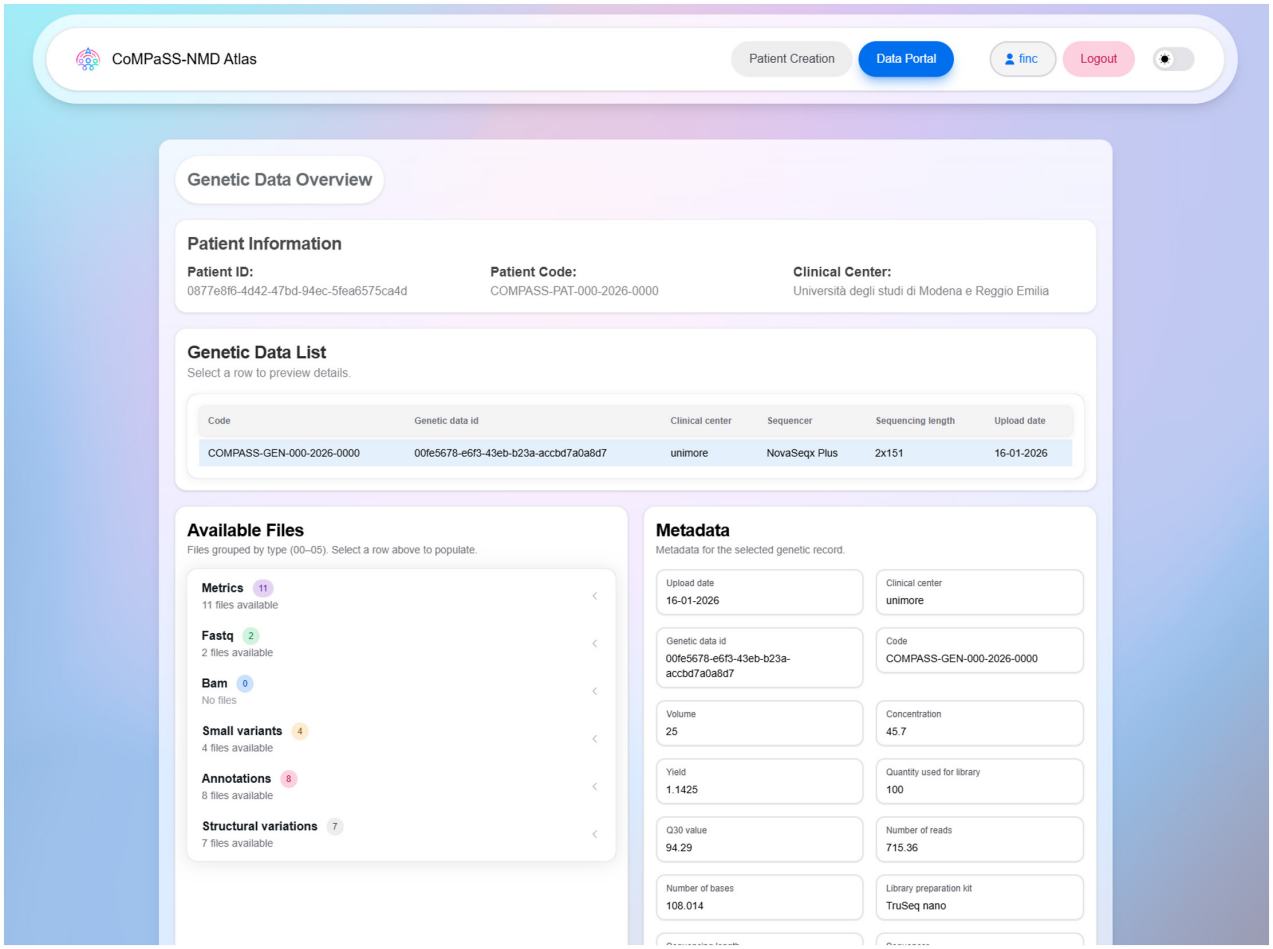
Genetic data module. Screenshot of the genetic data overview, showing raw sequencing outputs (FASTQ), variant files (VCF), and associated metadata.

## 3 Results

### 3.1 Data Exploration and Cohort Identification

A central exploration area lets authorized users browse and filter the patient collection. Because HPO is organized through parent–child “is‐a” relationships, searches retrieve not only a specific sign but broader categories and all descendant terms—e.g., a parent term for gait disturbance or shoulder‐girdle abnormality returns patients annotated with more specific subordinate features. Users navigate the ontology, combine multiple filters, and apply logical operators (AND, OR, NOT) to define cross‐domain cohorts spanning clinical, MRI, histopathological, and genetic data. Filtering combines structured criteria (center, onset age, sex) with HPO‐based features (e.g., proximal weakness, limited shoulder abduction). HPO is a global standard for computational phenotype encoding supporting semantic‐similarity and ML‐based inference across rare‐disease cohorts [7]; as of 2024 it holds over 17,000 terms and aligns with the GA4GH Phenopacket Schema [7]. This moves users from a broad overview to targeted cohorts and underpins the project's downstream AI‐supported stratification based on shared multimodal profiles rather than any single dimension (Figure 5). These capabilities are research‐support tools for hypothesis generation, not clinical diagnoses, and do not replace the treating physician's judgment. Technical detail on the RDF model and SPARQL queries is provided in Box 1.

**FIGURE 5:**
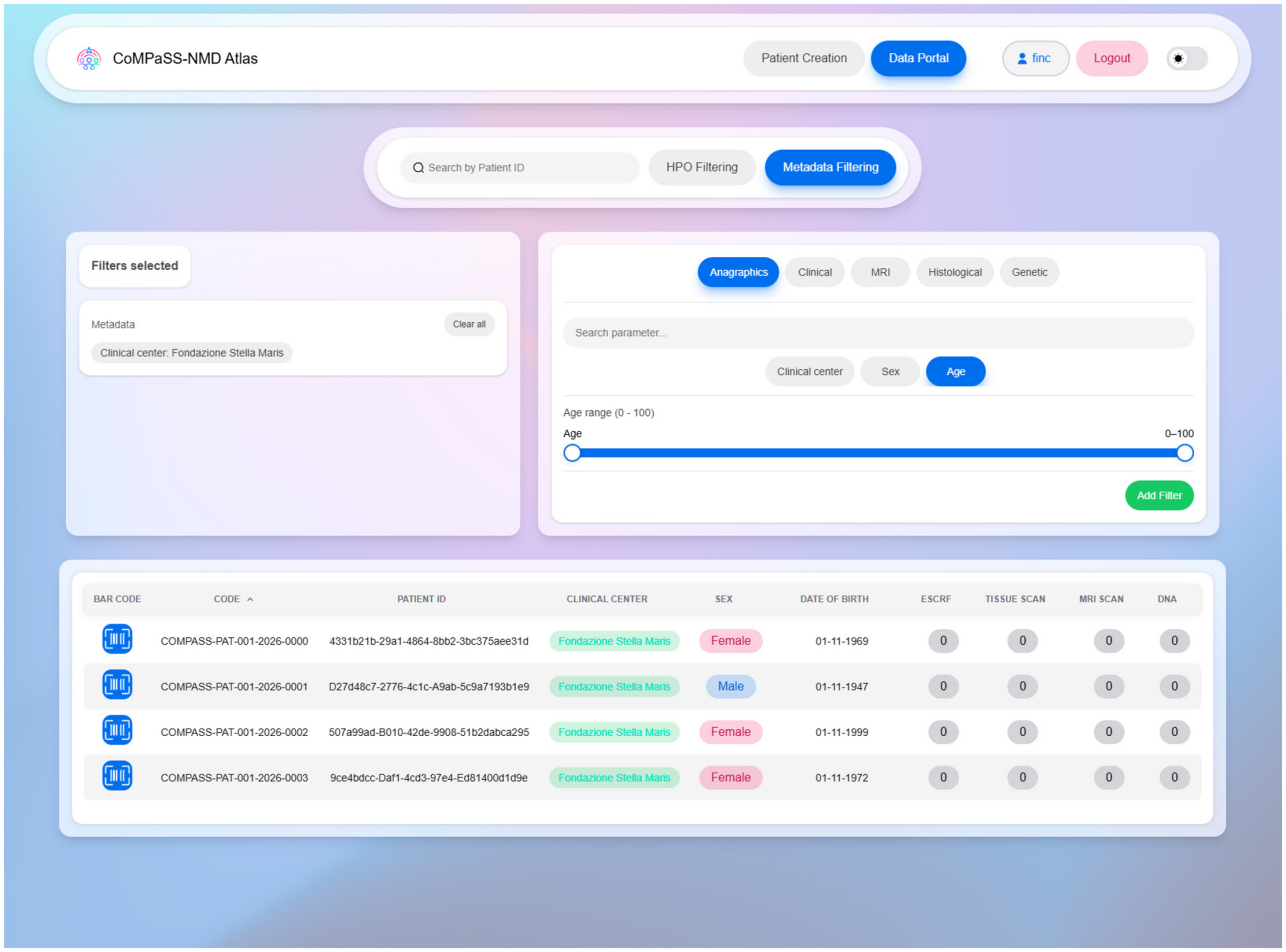
Data exploration portal. Screenshot of the cohort‐exploration interface with metadata and Human Phenotype Ontology (HPO) filtering. Dates of birth are displayed with the day set to 01, because only month and year of birth are stored.

### 3.2 Interoperability and FAIR Alignment

The Atlas was designed in alignment with the FAIR principles [2, 8]. Findability derives from standardized metadata and phenotype‐based filtering; Accessibility from authenticated, authorized interfaces, controlled downloads, and exportable metadata reports; Interoperability from REST APIs, standardized schemas, consistent metadata, and ontology‐based terms; and Reusability from preserving raw data in its original formats, exporting metadata, and maintaining traceable patient–data links. This alignment is especially critical in rare‐disease research, where data are limited and fragmented [9].

### 3.3 Security and Privacy

The platform manages pseudonymized data in accordance with the General Data Protection Regulation (GDPR); data were collected under protocols approved by local ethics committees, with written informed consent covering computational stratification and AI research. Directly identifying data (name, address, health number) are not held, whereas month and year of birth are retained as a clinical variable (Section 2.3); authentication uses Keycloak and authorization uses role‐ and attribute‐based rules. Large files reside in object storage, and structured metadata is stored in a relational database, allowing download permissions to be managed separately from metadata viewing and editing, with support for logging and auditing. Data governance runs through a centralized DAC: access is not granted by default but only upon a formal request specifying the scientific objective and ethical compliance, thereby decoupling metadata viewing from raw‐file download. The DAC is operational and comprises representatives of the contributing clinical centers, the technical and data‐management partners, an expert in ethics and data protection, and a patient representative. Applicants submit a written request stating the scientific objective, the data types and cohort required, evidence of ethics approval, and the planned data‐protection measures. Requests are assessed against four criteria—scientific merit, compatibility with the participants' informed consent, ethical and legal compliance, and data minimization—and are decided by majority vote after consultation of the contributing centers concerned. Decisions are documented; approved access is time‐limited, covered by a data‐use agreement, and logged by the platform.

### 3.4 Implementation Status and Technical Verification

The Atlas is deployed in production and is the system used for prospective data entry in the registered study (NCT06734949) [4]. Each module was verified in the development environment before release and again after promotion to production. Verification comprised (i) end‐to‐end functional tests of patient registration, clinical and histopathology form entry, MRI, histopathology, and sequencing uploads, record review, and export, using test records; (ii) access‐control tests confirming that Clinical Centre Contributors cannot view or edit records of another center and that Researcher accounts cannot upload data or download raw files without DAC authorization; (iii) transfer tests with files of up to tens of gigabytes through the presigned‐URL upload route, including verification of the link between each file and its pseudonymized record; and (iv) checks of audit logging for data‐entry, editing, and download events. Issues reported by clinical users during onboarding were tracked and resolved in successive releases.

## 4 Discussion

The Atlas addresses a core need for multimodal AI‐approachable projects: harmonized, secure, traceable data collection across distributed sites. Because HNMDs are difficult to classify from any single data source, linking clinical, genetic, pathological, and imaging data to a single pseudonymized record is intended to counter the fragmentation that leads to partial views and delayed or incomplete diagnoses. Modules for registration, clinical entry, MRI, histopathology, genetics, and cross‐patient exploration form an integrated environment in which heterogeneous data are progressively standardized, connected, and queried around the same patient. Separating structured metadata from a dedicated large‐file repository scales to diverse data, and the modular architecture supports future analytics, quality control, and AI components. HPO mapping anchors phenotypes to an internationally recognized standard, supporting genotype–phenotype analysis and interoperability with external systems.

The combined RBAC/ABAC model is essential for European multicenter work, balancing collaboration with privacy, governance, and center‐level data boundaries. The DAC (Section 3.3) evaluates raw‐data requests and decouples metadata exploration from raw‐file downloads, keeping access controlled, auditable, and aligned with all centers' ethical frameworks; it currently serves consortium‐internal requests, and opening the procedure to external researchers is the next governance milestone. Preserving raw data alongside structured metadata supports reproducibility and reuse, while HPO‐based hierarchical filtering links data collection to cohort discovery—identifying patients with similar characteristics even when features are recorded at different granularities. AI‐driven stratification is especially important for rare diseases, where small, fragmented datasets pose regulatory and technical challenges [10]. Alongside this centralized infrastructure, the project also explored federated learning to enable collaborative AI without centralizing data, aligning with initiatives such as the EURO‐NMD Registry Hub [9] and RD‐Connect [11]. The Atlas is expected to evolve toward greater pipeline interoperability, harmonized metadata, quality‐control metrics, improved auditing, and AI‐based clustering and patient‐similarity studies, serving as a semantic discovery environment for research on diagnosis, prognosis, and trial readiness. Ongoing user feedback will guide refinement.

### 4.1 Relation to Existing Registries and Platforms

The Atlas complements rather than duplicates existing neuromuscular data resources (Table 1). The EURO‐NMD Registry collects data on all neuromuscular patients seen at the network's expert centers in three layers of data elements—European Commission‐mandated, cross‐neuromuscular, and disease‐specific—and connects to other FAIR registries through the EURO‐NMD Registry Hub for federated analysis [9]. The TREAT‐NMD Global Registry Network links national, disease‐specific registries built on agreed core datasets, primarily for trial feasibility and recruitment [12]. The RD‐Connect Genome‐Phenome Analysis Platform provides standardized processing and collaborative analysis of exome and genome data linked to HPO‐coded phenotypes across all rare diseases [11, 13]. These resources prioritize breadth, epidemiology, care, and trial readiness, and capture imaging and histopathology, where included, as structured data elements. The Atlas addresses a different need: a research dataset of limited breadth but high depth, in which the primary data objects themselves—MRI series, histopathology tissue scans, and sequencing files—are stored with an HPO‐mapped clinical record under a single pseudonym, so that image‐, sequence‐, and phenotype‐derived features can be analyzed jointly by AI models. Its additional value therefore lies in (i) per‐patient linkage of raw multimodal files, (ii) a structured, multi‐reviewer histopathology form, (iii) ontology‐aware cross‐modal cohort queries, and (iv) prospective collection under a single protocol designed for model training. Because the Atlas uses HPO, ORPHA codes, and standard file formats, its metadata can in principle be mapped to the EURO‐NMD data elements and made discoverable to federated infrastructures; it is not intended to replace them.

**TABLE 1:** Positioning of the CoMPaSS‐NMD Atlas Platform relative to existing neuromuscular registries and rare‐disease platforms.

Feature	CoMPaSS‐NMD Atlas	EURO‐NMD Registry and Registry Hub [9]	TREAT‐NMD Global Registry Network [12]	RD‐Connect GPAP [11, 13]
Primary purpose	Multimodal research dataset for AI‐based stratification and diagnosis	Registry of patients seen at expert centers: epidemiology, care, cohort finding, trial readiness	Trial feasibility and recruitment; care and natural‐history data	Genomic diagnosis and gene discovery
Disease scope	Hereditary NMDs	All NMDs covered by EURO‐NMD	NMDs with disease‐specific registries (e.g., DMD, SMA)	All rare diseases
Data entry	Clinicians at contributing centers (eCRF, eHCRF)	Expert centers; patient‐reported outcomes	Patient‐ and/or clinician‐reported, by national registry	Submitting clinicians and researchers
Clinical data	About 125 core items, HPO‐mapped	Three layers of data elements (EU, cross‐NMD, disease‐specific)	Disease‐specific core datasets	HPO‐coded phenotypes
Imaging	Raw MRI series (DICOM, NIfTI) with metadata (eMRF)	Structured data elements	Not a core component	Not included
Histopathology	Tissue‐scan images plus structured multi‐reviewer form (eHCRF)	Structured data elements	Not a core component	Not included
Genetics	Raw FASTQ and VCF files with metadata (eGRF)	Structured data elements	Reported genetic diagnosis	Exome/genome data with standardized processing and variant analysis
Architecture	Central repository (relational database and object store)	Registry linked to a federated, FAIR Registry Hub	Network of independent national registries	Central analysis platform
Access	Role‐ and center‐based; raw files via DAC	Registry governance; federated queries via Hub	Enquiries through network governance	Registered, authorized users

*Note:* Characteristics of external resources are summarized from the cited publications and may have evolved since.

Abbreviations: AI, artificial intelligence; DAC, Data Access Committee; DMD, Duchenne muscular dystrophy; eCRF, electronic clinical case report form; eGRF, electronic genetic case report form; eHCRF, electronic histopathology case report form; eMRF, electronic MRI case report form; GPAP, Genome‐Phenome Analysis Platform; HPO, Human Phenotype Ontology; MRI, magnetic resonance imaging; NMD, neuromuscular disorder; SMA, spinal muscular atrophy.

### 4.2 Rationale for Reporting the Platform at This Stage

We report the platform separately from its clinical validation for three reasons. First, the architecture, data model, pseudonymization scheme, and access‐control model are implemented and in use (Section 3.4), whereas the ongoing validation study addresses different questions—data completeness at scale, inter‐rater agreement, harmonization effectiveness, and the performance of AI‐based stratification—whose results will be reported separately. Second, these forthcoming clinical and analytical reports require a citable, peer‐reviewed description of how the data were collected, structured, and governed. Third, early disclosure allows other networks to scrutinize the design and to align data elements while collection is still ongoing. We expect validation to lead to refinements of form items, HPO mappings, and quality‐control rules rather than to changes in architecture; such modifications will be versioned and reported together with the validation results. Other infrastructure descriptions, such as that of the EURO‐NMD Registry [9], were likewise published when clinical use was only beginning.

### 4.3 Limitations

This manuscript describes the design, implementation, and technical verification of the platform, not a completed clinical validation study; usability, data completeness at scale, diagnostic impact, harmonization effectiveness, and AI‐stratification performance remain outside its scope while data collection continues. Despite structured forms, HPO mapping, and standardized pathology reporting, some inter‐rater variability cannot be ruled out in interpretive assessments, and formal inter‐rater evaluation is ongoing. The HPO's evolving releases require periodic review of entries recorded under earlier versions. Finally, cross‐border constraints on transferring and storing sensitive data—particularly imaging—may limit access to raw files even after DAC approval, reflecting the evolving European health‐data landscape. Because month and year of birth are retained, the dataset is pseudonymized rather than anonymized, and in rare diseases the combination of month and year of birth, center, and genotype carries a residual re‐identification risk; exports to users outside the contributing center will therefore be restricted to derived age variables. A structured usability study with independent test users has not yet been performed.

## 5 Conclusions

The CoMPaSS‐NMD Atlas Platform provides a secure, modular, and scalable system for collecting pseudonymized multimodal data on hereditary neuromuscular disorders. It links clinical, MRI, histopathology, and genetic data within traceable patient records to build datasets for future AI‐ and ML‐based stratification. By combining modern web technologies, structured metadata management, scalable object storage, secure identity handling, and fine‐grained access control—and operating within an established network of specialist centers—it offers a FAIR‐aligned foundation for data exploration, cohort selection, and, once validated, precision‐diagnosis research in neuromuscular diseases.

## Author Contributions

**R. Corrias:** software, formal analysis, writing – review and editing, supervision, validation, methodology. **P. Foszner:** software, formal analysis, data curation, supervision, resources, writing – review and editing, validation, methodology. **A. Nuredini:** data curation, validation, supervision, writing – review and editing. **D. Mendelsohn:** writing – review and editing, validation, data curation. **A. Töpf:** methodology, conceptualization, data curation, supervision, formal analysis, validation, funding acquisition, writing – review and editing. **J. Díaz‐Manera:** conceptualization, supervision, data curation, formal analysis, writing – review and editing, methodology, validation, funding acquisition. **M. Obach:** conceptualization, methodology, data curation, supervision, resources, formal analysis, validation, writing – review and editing. **A. Garzo:** conceptualization, methodology, data curation, writing – review and editing. **M. Savarese:** conceptualization, methodology, data curation, supervision, formal analysis, validation, resources, writing – review and editing, funding acquisition. **J. Verdú‐Díaz:** data curation, software, formal analysis, methodology, validation, visualization, writing – review and editing. **S. Pini:** data curation, validation, writing – review and editing. **G. La Vella:** data curation, validation, writing – review and editing. **N. Albano:** data curation, validation, writing – review and editing. **M. Markiel:** data curation, validation, writing – review and editing. **L. Piorecki:** methodology, software, data curation, supervision, formal analysis, writing – review and editing. **A. Suwalska:** data curation, software, validation, formal analysis, writing – review and editing. **M. Socha:** data curation, validation, writing – review and editing. **M. Marczyk:** methodology, software, data curation, formal analysis, writing – review and editing. **S. Bonelli:** writing – review and editing, validation. **A. DeAngelis:** validation, writing – review and editing. **J. Laporte:** conceptualization, methodology, data curation, supervision, formal analysis, validation, funding acquisition, resources, writing – review and editing. **G. Caudullo:** methodology, data curation, formal analysis, validation, writing – review and editing. **E. Savino:** validation, writing – review and editing, data curation. **V. Straub:** conceptualization, methodology, data curation, supervision, writing – review and editing, funding acquisition. **F. M. Santorelli:** conceptualization, methodology, data curation, supervision, writing – review and editing, funding acquisition. **J. Polanska:** methodology, conceptualization, funding acquisition, validation, software, formal analysis, data curation, supervision, resources, writing – review and editing. **M. P. Scipioni:** validation, writing – review and editing. **R. Tupler:** conceptualization, methodology, data curation, supervision, formal analysis, funding acquisition, project administration, writing – review and editing, writing – original draft. **B. Schoser:** conceptualization, funding acquisition, writing – original draft, writing – review and editing, visualization, validation, methodology, formal analysis, project administration, supervision, data curation, resources, investigation.

## Funding

This work was developed within the CoMPaSS‐NMD project, “Computational Models for New Patient Stratification Strategies of Neuromuscular Disorders,” funded by the European Union under the Horizon Europe Programme, grant agreement number 101080874. Views and opinions expressed are, however, those of the author(s) only and do not necessarily reflect those of the European Union or the European Commission. Neither the European Union nor the granting authority can be held responsible for them. F. M. Santorelli is supported by the Italian Ministry of Health (Ricerca Corrente 2025).

## Ethics Statement

This manuscript describes a technical platform for the pseudonymized management of biomedical data. Collection and use of patient‐related data within the CoMPaSS‐NMD project comply with applicable ethical approvals, informed‐consent procedures, institutional governance rules, and European and national data‐protection regulations. The technical development described was carried out within the framework of the CoMPaSS‐NMD project to manage the personal data collected in that project. The protocols for data collection were supervised and approved by the Ethics Committee of Area Vasta Emilia Nord (project number 101080874), the Ethics Committee of Ludwig‐Maximilians‐Universität München (project number 24‐0242), and the Ethics Committee of Regione Toscana.

## Conflicts of Interest

The authors declare no conflicts of interest.

## Supporting information


**Video S1:** Narrated walkthrough of the CoMPaSS‐NMD Atlas Platform (5 min 55 s), following a clinical use case: pseudonymized registration of a new patient with automatic generation of the CoMPaSS Code and barcode; cohort search using Human Phenotype Ontology (HPO) and clinical‐metadata filters (steppage gait, inability to walk on heels, ptosis, extraocular muscle weakness, dysphagia, dilated cardiomyopathy, elevated creatine kinase); review of a matching patient's clinical form, including neurological examination and Medical Research Council scores; and access to the linked histopathology, MRI, and genetic data. All records shown are test records; no patient data are displayed. A transcript of the narration is provided.

## Data Availability

No new patient‐level datasets are presented in this manuscript. The article describes the design and implementation of the CoMPaSS‐NMD Atlas Platform. Data availability for the wider CoMPaSS‐NMD project is subject to project governance, ethical approvals, and applicable European and national data protection regulations.

## References

[1] European Commission , “Computational Models for New Patient Stratification Strategies in Neuromuscular Disorders— oMPaSS‐NMD,” CORDIS EU Research Results. Grant Agreement ID: 101080874, accessed May 8, 2026, https://cordis.europa.eu/project/id/101080874.

[2] A. Nuredini , M. Savarese , F. M. Santorelli , and R. G. Tupler , “Empowering Clinicians With Artificial Intelligence in Hereditary Neuromuscular Disorders: Training the Next Generation Through the CoMPaSS‐NMD Young Investigator Training Program and the CoMPaSS‐NMD Autumn School Workshop Report,” Acta Myologica 44, no. 2 (2025): 62–66, 10.36185/2532-1900-927. PMC12250589 40626680

[3] CoMPaSS‐NMD Consortium , “Resources,” accessed May 8, 2026, https://compass‐nmd.eu/resources/.

[4] ClinicalTrials.gov , “Computational Models for New Patient Stratification Strategies of Neuromuscular Disorders (CoMPaSS‐NMD),” ClinicalTrials.gov identifier: NCT06734949, accessed May 8, 2026, https://clinicaltrials.gov/study/NCT06734949.

[5] S. Köhler , M. Gargano , N. Matentzoglu , et al., “The Human Phenotype Ontology in 2021,” Nucleic Acids Research 49, no. D1 (2021): D1207–D1217, 10.1093/nar/gkaa1043. PMC7778952 33264411

[6] B. Udd , W. Stenzel , A. Oldfors , et al., “1st ENMC European Meeting: The EURO‐NMD Pathology Working Group Recommended Standards for Muscle Pathology, Amsterdam, The Netherlands, 7 December 2018,” Neuromuscular Disorders 29, no. 6 (2019): 483–485, 10.1016/j.nmd.2019.03.002. 31101462

[7] M. A. Gargano , N. Matentzoglu , B. Coleman , et al., “The Human Phenotype Ontology in 2024: Phenotypes Around the World,” Nucleic Acids Research 52, no. D1 (2024): D1333–D1346, 10.1093/nar/gkad1005. PMC10767975 37953324

[8] M. D. Wilkinson , M. Dumontier , I. J. Aalbersberg , et al., “The FAIR Guiding Principles for Scientific Data Management and Stewardship,” Scientific Data 3 (2016): 160018, 10.1038/sdata.2016.18. PMC4792175 26978244

[9] A. Atalaia , D. Wandrei , N. Lalout , et al., “EURO‐NMD Registry: Federated FAIR Infrastructure, Innovative Technologies and Concepts of a Patient‐Centred Registry for Rare Neuromuscular Disorders,” Orphanet Journal of Rare Diseases 19, no. 1 (2024): 66, 10.1186/s13023-024-03059-3. PMC10865673 38355534

[10] J. Wang and F. Ma , “Federated Learning for Rare Disease Detection: A Survey,” Rare Diseases and Orphan Drugs Journal 2, no. 4 (2023): 22, 10.20517/rdodj.2023.16.

[11] R. Thompson , L. Johnston , D. Taruscio , et al., “RD‐Connect: An Integrated Platform Connecting Databases, Registries, Biobanks and Clinical Bioinformatics for Rare Disease Research,” Journal of General Internal Medicine 29, no. Suppl 3 (2014): S780–S787, 10.1007/s11606-014-2908-8. PMC4124112 25029978

[12] C. L. Bladen , K. Rafferty , V. Straub , et al., “The TREAT‐NMD Duchenne Muscular Dystrophy Registries: Conception, Design, and Utilization by Industry and Academia,” Human Mutation 34, no. 11 (2013): 1449–1457, 10.1002/humu.22390. 23913485

[13] S. Laurie , D. Piscia , L. Matalonga , et al., “The RD‐Connect Genome‐Phenome Analysis Platform: Accelerating Diagnosis, Research, and Gene Discovery for Rare Diseases,” Human Mutation 43, no. 6 (2022): 717–733, 10.1002/humu.24353. PMC9324157 35178824

